# Integrated Chemical and Biological Evaluation of Linden Honeydew Honey from Bosnia and Herzegovina: Composition and Cellular Effects

**DOI:** 10.3390/foods14101668

**Published:** 2025-05-08

**Authors:** Ana Barbarić, Lara Saftić Martinović, Zvonimir Marijanović, Lea Juretić, Andreja Jurič, Danijela Petrović, Violeta Šoljić, Ivana Gobin

**Affiliations:** 1 Faculty of Health Studies, University of Mostar, Zrinskog Frankopana 34, 88000 Mostar, Bosnia and Herzegovina; ana.planinic@fzs.sum.ba (A.B.); danijela.petrovic@aptf.sum.ba (D.P.); violeta.soljic@fzs.sum.ba (V.Š.); 2 Faculty of Medicine, University of Rijeka, Brace Branchetta 20, 51000 Rijeka, Croatia; lea.juretic@uniri.hr (L.J.); ivana.gobin@medri.uniri.hr (I.G.); 3 Faculty of Biotechnology and Drug Development, University of Rijeka, Radmile Matejčić 2, 51000 Rijeka, Croatia; 4 Department of Food Technology and Biotechnology, Faculty of Chemistry and Technology, University of Split, Ruđera Boškovića 35, 21000 Split, Croatia; zvonimir.marijanovic@ktf-split.hr; 5 Division of Toxicology, Institute for Medical Research and Occupational Health, 10000 Zagreb, Croatia; ajuric@imi.hr; 6 Faculty of Agriculture and Food Technology, University of Mostar, Biskupa Čule bb, 88000 Mostar, Bosnia and Herzegovina; 7 Laboratory of Morphology, Department of Histology and Embryology, School of Medicine, University of Mostar, 88000 Mostar, Bosnia and Herzegovina; 8 Teaching Institute of Public Health of Primorje-Gorski Kotar County, Krešimirova 52a, 51000 Rijeka, Croatia

**Keywords:** linden honeydew honey, *Tilia* spp., physicochemical properties, antioxidant activity, antimicrobial activity, volatile compounds, phenolic profile, HaCaT cells, wound healing, cytotoxicity

## Abstract

Honeydew honey (HH) is a distinctive type of honey known for its dark colour, high mineral and polyphenol content, and pronounced biological activity. This study continues previous research on beech and chestnut honeydew honeys by presenting a comprehensive analysis of linden honeydew honey (LHH) from Bosnia and Herzegovina—a variety that, until now, has not been characterised in detail. Physicochemical parameters confirmed its classification as HH, with high electrical conductivity (1.21 mS/cm) and low moisture (15.1%). GC-MS analysis revealed a unique volatile profile dominated by α-terpinolene (17.4%), distinguishing LHH from other HH types. The sample exhibited high total phenolic content (816.38 mg GAE/kg) and moderate antioxidant capacity (1.11 mmol TE/kg). Antimicrobial testing demonstrated strong activity against *Staphylococcus aureus* and *Methicillin-resistant Staphylococcus aureus* (MRSA), with lower efficacy against Gram-negative bacteria. No cytotoxic effects were observed in HaCaT keratinocytes at concentrations up to 60 mg/mL, and wound healing assays showed improved scratch closure reaching approximately 30% after 24 h and 41% after 48 h compared to the control. These results indicate that LHH possesses promising bioactive properties and potential for dermatological application. Further studies with broader sample sets are needed to explore variability and confirm the therapeutic relevance of LHH in comparison to other honeydew types.

## 1. Introduction

Honey is a natural product with a complex composition, recognised for its nutritional value and health-related properties, such as antibacterial, antioxidant, and anti-inflammatory effects, as well as its potential role in supporting wound healing and treating infections [1,2]. While nectar honey has been extensively studied, there has been growing interest in honeydew honey (HH), a distinct category that differs in origin and composition. Unlike nectar honey, HH does not originate from floral nectar, but rather from the sweet secretions of sap-feeding insects, such as aphids, that extract nutrients from plant phoem. The chemical composition of these insect-produced secretions (honeydew) significantly depends on the plant species serving as the insect’s food source, the specific insect species involved, and various environmental conditions that affect both plant physiology and insect metabolism, particularly temperature and water availability [3,4]. The production and quality standards of honey in the European Union are regulated by Council Directive 2001/110/EC, which defines requirements for honeydew and other types of honey [5].

HH is characterised by its darker colour, which may include a specific greenish hue in the case of pure fir honeydew [6]. Its chemical composition is notable for its high concentrations of minerals, organic acids, enzymes, proteins, amino acids, and vitamins, contributing to its unique nutritional and bioactive profile [4,7]. HH also contains significant amounts of polyphenols and flavonoids, such as protocatechuic acid, *p*-hydroxybenzoic, caffeic, *p*-coumaric and ferulic acids, and flavonoids such as chrysin and pinocembrin, which contribute to its antioxidant and antibacterial properties [4,8,9,10,11,12,13,14,15]. As HH gains increasing popularity among consumers and the food industry, its identification and certification are becoming more important. This honey is distinguished by its specific physicochemical and sensory properties, including a higher pH value, high electrical conductivity, elevated ash content, and a mild resinous aroma [16]. Due to its pronounced and complex taste, along with its high bioactive compound content, honeydew honey has significant potential for applications in the medical and food industries. Although current regulations do not categorise honeydew honeys based on botanical origin [5], our recent study [17] has demonstrated that honeydews from different plant sources can exhibit distinct chemical and biological profiles.

Motivated by these findings, we aimed to investigate linden honeydew honey (LHH)—a rare and understudied honey variety—in order to assess whether it possesses a unique composition and bioactivity profile comparable to or distinct from other types of HH. Linden (*Tilia* spp.) is a melliferous species whose nectar honey has been widely studied for its distinctive aroma and biological properties, including antioxidant and antimicrobial effects. Numerous studies have characterised the volatile and phenolic composition of linden nectar honey, identifying unique compounds such as lindenin, methyl syringate, and terpene glycosides [18,19,20,21]. These compounds have been recognised as reliable markers of botanical origin and honey authenticity. Traditionally, linden nectar honey has been used to treat respiratory and inflammatory conditions. Studies have shown that it contains high concentrations of phenolic and flavonoid compounds, including gallic acid, *p*-hydroxybenzoic acid, lindenin, and cis- and trans-abscisic acid, all of which contribute to its potent antioxidant potential [19,22]. Its historical application in treating respiratory infections is further supported by its antibacterial activity against *Staphylococcus aureus* and *Staphylococcus epidermidis* [18].

However, despite the well-documented composition and bioactivity of linden nectar honey, the chemical profile and therapeutic potential of linden honeydew honey is still lacking. Considering this gap in the literature, LHH represents a particularly interesting candidate for further investigation, particularly in the context of potential therapeutic applications. Moreover, the area of Bosnia and Herzegovina, with its rich flora and favourable climatic conditions, provides an ideal environment for the production of high-quality HH. While honey samples from Bosnia and Herzegovina have been the subject of several studies, the majority have focused on nectar honeys. Honeydew honeys remain scarcely represented, and even in cases where their physicochemical or biological properties were assessed, the botanical origin of the honeydew was not characterised, leaving a significant gap in the understanding of these rare honeys [23,24,25,26]. Local environmental factors contribute to the development of honey with unique features, including mineral richness, a complex flavour profile, and a high content of bioactive compounds. These natural advantages make the region particularly suitable for the collection of rare unifloral honeydew varieties, such as LHH, and add further value to the study [17].

In this study, we aimed to comprehensively characterise the physicochemical and bioactive properties of LHH, with particular focus on its antimicrobial activity, antioxidant potential, and effects on human keratinocytes (HaCaTs) in vitro, including cell viability and wound healing assays. Given the increasing recognition of honeydew honey as a functional food and its potential applications in dermatology, these biological endpoints are of particular relevance. Special emphasis was placed on evaluating the cytocompatibility of LHH with HaCaT cells and its capacity to stimulate wound closure, as linden-derived compounds are known to exhibit a range of bioactive effects, particularly anti-inflammatory and tissue-regenerative properties [27,28]. The physicochemical and antimicrobial results obtained for LHH will be directly compared to our previously published findings on chestnut and beech honeydew honeys in order to identify both shared and unique features across different botanical sources. Furthermore, the newly introduced assays assessing cell viability and in vitro wound healing are discussed in the context of the existing research on both honeydew and nectar honeys, thereby contributing to a broader understanding of the biological activities associated with LHH. By integrating physicochemical profiling with antimicrobial, antioxidant, and cellular-level analyses, this study provides a more comprehensive insight into the multifaceted properties of LHH.

## 2. Materials and Methods

### 2.1. Honey Sample

**Figure 1:**
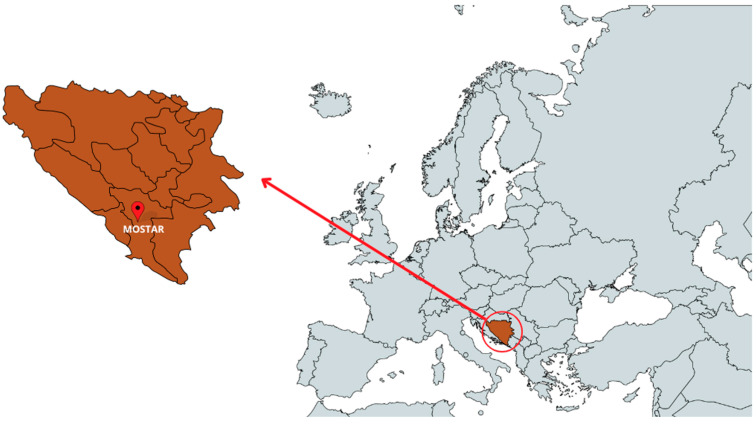
Map of Bosnia and Herzegovina showing the location where linden honeydew honey (LHH) sample was collected.

A representative sample of linden honeydew honey (LHH) was collected in 2023 directly from beekeepers in Mostar, Bosnia and Herzegovina (43°20′58″ N, 17°48′45″ E) (Figure 1). The LHH was obtained in a sterile glass container to prevent contamination and was stored at 4 °C in a dark environment to preserve its physical, chemical, and microbiological characteristics until analysis [29] Codex Alimentarius, 2001).

### 2.2. Characterisation and Authentication of Linden Honeydew Honey Origin

#### 2.2.1. Physicochemical Analysis

The physicochemical properties of a representative LHH sample were analysed following the Regulation on Methods for Control of Honey and Other Bee Products (Federation of Bosnia and Herzegovina, 2009) [30].

##### Water Content

The water content in the LHH sample was measured using the refractometric method at a temperature of 20 °C. A correction factor of ±0.00023 per °C was applied for any measurements taken at different temperatures. The final water content (% *w*/*w*) was determined using standard correlation tables for refractive indices.

##### Electrical Conductivity

Electrical conductivity was evaluated to determine the botanical origin. A 20 g sample was dissolved in 100 mL of distilled water, and measurements were taken with a conductometer (WTW, Weilheim, Germany) at 20 °C. Any deviations from this temperature were adjusted using a standard correction factor of 3.2% per °C [31].

##### Determination of Free Acidity

The acidity was determined by dissolving 10 g of honey in 75 mL of carbon dioxide-free water. The mixture was stirred using a magnetic stirrer, and the initial pH was measured by immersing the pH electrode into the solution. The sample was then titrated with 0.1 M sodium hydroxide (NaOH) until the pH reached 8.30, with the reading taken within 2 min from the start of titration. The total acidity was calculated by multiplying the volume of NaOH consumed (in millilitres) by a factor of 10 following the method described previously [17].

#### 2.2.2. Colour Analysis

The colour of the honey sample was measured using a Lovibond Honey ColorPod comparator (Lovibond, London, UK). The transmittance of the honey was determined at 430 nm and 530 nm, and the results were expressed on the Pfund scale [32].

#### 2.2.3. Pollen Analysis

A 10 g LHH sample was dissolved in 20 mL of distilled water, heated to 40 °C to dissolve the sugars, and then centrifuged at 1375× *g* (3500 rpm) for 15 min. The sediment was placed on glass slides, dried at a temperature of ≤40 °C, and embedded in glycerine jelly for microscopic examination. At least 300 pollen grains per sample were identified using morphological characteristics such as size, shape, and exine structure [33].

#### 2.2.4. Analysis of Volatile Compounds

Volatile compounds were extracted by headspace solid-phase microextraction (HS-SPME), and subsequently analysed using gas chromatography with flame ionisation detection (GC-FID) and gas chromatography coupled to mass spectrometry (GC-MS) [34]. Polydimethylsiloxane/divinylbenzene (PDMS/DVB) fibre was used. Prior to the analysis, the fibre was thermally conditioned at 150 °C for 24 h in order to remove potential contaminants and ensure the reliability of the extraction. A total of 5 mL of LHH solution (1:1 *v*/*v* with saturated NaCl solution) was placed in a sealed 15 mL vial and subjected to HS-SPME extraction at 60 °C. The sample was equilibrated for 15 min and extracted for 45 min under continuous stirring at 1000 rpm. The SPME fibre was then thermally desorbed in the GC injector (250 °C). The GC-FID analysis was performed on an Agilent 7890A gas chromatograph (Agilent Technologies, Inc., SantaClara, CA, USA) with an HP-5MS capillary column using helium (1.0 mL/min) as the carrier gas. The oven temperature was programmed from 70 °C (2 min) to 200 °C at 3 °C/min, followed by a 15 min hold at 200 °C. The GC-MS analysis was conducted on an Agilent 7820A gas chromatograph coupled with a 5977E mass selective detector (Agilent Technologies, Inc., Santa Clara, CA, USA; EI mode, 70 eV, scan range 30–300 amu) under identical chromatographic conditions [35].

Volatile compounds were identified by comparing their retention indices (RIs) to *n*-alkane standards (C9–C25) and matching mass spectra with reference compounds from the Wiley 14 and NIST 09 libraries. Relative abundances were determined from GC peak areas using the normalisation method, with average values calculated from duplicate GC-FID and GC-MS analyses.

### 2.3. Biological Activity

#### 2.3.1. Antimicrobial Activity

The antimicrobial activity of LHH was evaluated using two standardised methods: the agar well diffusion assay and the broth dilution method. These techniques enable the precise assessment of the inhibitory effect of the sample against selected bacterial strains and were conducted in accordance with the guidelines of the European Committee on Antibacterial Susceptibility Testing (EUCAST) [36].

The standard laboratory strains used in the study were *Acinetobacter baumannii* (ATCC 19606 and ATCC BAA–1605), *Staphylococcus aureus* ATCC 25923, *Pseudomonas aeruginosa* ATCC 27853, *Escherichia coli* ATCC 25922, *Enterococcus faecalis* ATCC 29212, *Bacillus cereus* 10876, *Salmonella* *typhimurium* ATCC 14028, and *Listeria monocytogenes* ATCC 19115 from the culture collection of the Department of Microbiology and Parasitology, University of Rijeka. The bacterial strains were stored at −80 °C in glycerol broth (10% glycerol) (Biolife, Milan, Italy).

For testing purposes, the bacteria were grown on Mueller–Hinton Broth (MHB) (Difco, Sparks, MD, USA) at 37 °C for 24 h with constant agitation at 120 rpm (Unimax 1010, Heidolph, Schwabach, Germany). The optical density of the bacterial suspension was additionally determined with a spectrophotometer at 550 nm (Eppendorf BioPhotometer, Hamburg, Germany), and the number of bacterial cells was extrapolated from the standard growth curve. A defined number of viable bacterial cells was used for the experiments, which was obtained by plating 10-fold dilutions onto blood agar (Biolife). After 24 h incubation at 37 °C, the number of bacterial colonies was determined as the number of colony-forming units (CFU/mL).

##### Agar Well Diffusion Assay

A bacterial suspension (10^8^ CFU/mL) was prepared and spread onto Mueller–Hinton agar plates. After aseptically drilling a well (6 mm in diameter) in the agar, 30 µL of LHH solution (0.4 g/mL) was added. The plates were then incubated overnight at 35 ± 2 °C. Antimicrobial activity was assessed by measuring the diameter of the resulting inhibition zones in millimetres [37].

##### Broth Dilution Method

Serial two-fold dilutions of LHH were prepared in Muller–Hinton broth, covering a concentration range of 0.0125 to 0.4 g/mL. Equal amounts of bacterial suspension (10^6^ CFU/mL) were added to the diluted LHH, and the mixtures were incubated for 24 h at 35 ± 2 °C. The minimum inhibitory concentration (MIC) was defined as the lowest concentration of LHH that completely inhibited visible bacterial growth. The minimum bactericidal concentration (MBC) was determined by plating the MIC samples on fresh agar and incubating for an additional 24 h [38].

#### 2.3.2. Phenolic Content and Antioxidant Activity

##### Determination of Total Phenolic Content (TPC)

The total phenolic content was assessed following a previously reported procedure [39]. In short, 100 µL of a 10% (*w*/*v*) aqueous solution of LHH was combined with 200 µL of 2 M Folin–Ciocalteu reagent and 1.4 mL of ultrapure water. After allowing the reaction mixture to stand for 5 min, 1.5 mL of a 6% (*w*/*v*) sodium carbonate solution was added. The samples were then incubated at 40 °C for 30 min, and the absorbance was subsequently measured at 760 nm. The TPC values were expressed as milligrams of gallic acid equivalents (GAE) per kilogram of honeydew honey.

##### Determination of Total Flavones/Flavonols Content (TFC)

The total content of flavones and flavonols was assessed using a slightly adapted procedure based on a previously reported method [17]. In brief, 400 µL of a 10% (*w*/*v*) aqueous solution of LHH was mixed with 700 µL of methanol, after which 100 µL of a 5% aluminium chloride solution prepared in methanol was added. The mixture was incubated at room temperature for 30 min, and the absorbance was subsequently measured at 415 nm. The amount of flavones and flavonols was expressed as milligrams of quercetin equivalents (QE) per kilogram of honeydew honey.

##### Determination of Total Flavanones/Dihydroflavonols Content

The total flavanones and dihydroflavonols content was assessed according to a previously described method [17]. In short, a 10% (*w*/*v*) aqueous solution of LHH was combined with 200 µL of 2,4-dinitrophenylhydrazine (97% purity), followed by incubation at 50 °C for 30 min. After cooling the reaction mixture to room temperature, 700 µL of a 10% potassium hydroxide solution prepared in methanol was added. Subsequently, 50 µL of the resulting solution was diluted with 2.450 mL of methanol, and the absorbance was recorded at 486 nm. The concentration of flavanones and dihydroflavonols was expressed as grams of naringenin equivalents (NAR) per kilogram of honeydew honey.

##### Determination of DPPH Radical Scavenging Activity

The 2,2-Diphenyl-1-picrylhydrazyl (DPPH) radical scavenging activity was measured using a slightly modified method described previously [40]. Briefly, an aliquot (150 µL) of 10% (*w*/*v*) LHH aqueous solution was mixed with 1.85 mL of methanol and 1.5 mL of DPPH methanolic solution (180 mol/L). The mixture was incubated in the dark at room temperature for 30 min, and the absorbance was measured at 517 nm. The results were expressed as mmol of Trolox equivalents (TE) per kg of honeydew honey.

#### 2.3.3. Assessment of Cytotoxicity (LC_50_)

Human keratinocyte cells (HaCaTs) derived from adult skin were maintained in 75 cm^2^ culture flasks using complete Dulbecco’s Modified Eagle’s Medium (DMEM). The culture medium was composed of DMEM (Sigma-Aldrich, Milan, Italy) supplemented with 10% heat-inactivated fetal bovine serum (FBS) and 1% L-glutamine (both Sigma-Aldrich, Milan, Italy). An EDTA-trypsin solution (Sigma-Aldrich, Milan, Italy) was used to separate the cells from the substrate, and the number of cells was determined by hemocytometry with Trypan Blue staining. In order to assess the viability, the LHH samples were previously dissolved in Mueller–Hinton broth (MHB) and diluted in DMEM to final concentrations of 2.5 mg/mL–60 mg/mL. After a 24 h incubation of the cells with the test samples, cell viability was assessed using the XTT assay according to a standard protocol [41].

#### 2.3.4. Wound Healing Assay

For the wound healing assay, HaCaT cells were grown in DMEM supplemented with 10% foetal bovine serum (FBS) and 0.1% antibiotics–antimycotics (penicillin, streptomycin, and amphotericin B). The cells were maintained under standard conditions at 37 °C and 5% CO_2_ until they reached 80–90% confluence. A total of 24 h before the experiment, the medium was removed and replaced with a serum-free medium. To simulate a wound, a sterile 200 µL pipette tip was used to make a straight-line scratch on the cells. After that, the cells were washed with Dulbecco’s phosphate-buffered saline (DPBS) to remove debris. A 0.1% honey solution was prepared, added to DMEM, and added to well plates at a volume of 2 mL per well. The control group was maintained in DMEM without the addition of honeydew honey. The cells were incubated for 24 and 48 h, and the wound healing process was documented using an inverted microscope (Olympus IX73, Olympus, Belgrade, Serbia) with a digital camera. Four representative images were taken in each test well within the marked scratch area. Quantification of the degree of wound healing was performed using the ImageJ software 1.8.0. (NIH, Bethesda, Rockville, MD, USA), and the percentage of wound closure was calculated according to the following formula:

Equation (1)

Wound closure (%) = (Area t_0_−Area t_24_ or t_48_)/Area t_0_ × 100. (1)

## 3. Results and Discussion

### 3.1. Physicochemical Properties

In order to assess the quality and authenticity of LHH, key physicochemical parameters such as water content, electrical conductivity, colour, free acidity, and pH were analysed. These characteristics not only enable the accurate classification of honey but also provide insight into its botanical and geographical origin. The results obtained from the analysis are shown in Table 1.

The physicochemical properties of honey play a pivotal role in determining its quality, stability, and potential shelf life. Among these, water content is particularly critical, as lower humidity reduces the risk of fermentation and crystallisation, thereby preserving the honey’s integrity over time [43]. International guidelines set a maximum permissible water content of 20%, with exceptions for certain varieties such as heather honey, which may contain up to 23% [44]. In the analysed LHH sample, the water content was found to be 15.10%, which aligns with quality standards. This value is consistent with previously reported moisture ranges for HH, typically between 15% and 18%, and is largely influenced by environmental factors such as climate, harvest timing, and storage conditions [43,44].

Electrical conductivity serves as another key differentiating factor between HHs and nectar honeys, primarily due to the higher mineral and organic acid content found in the honeydew varieties. A conductivity value above 0.8 mS/cm is indicative of HH [45]. In our sample, LHH exhibited a conductivity of 1.21 mS/cm, confirming its classification as HH. This value aligns with the typical range for HH (0.8–1.65 mS/cm), while floral honeys generally show lower conductivity values, ranging from 0.5 to 0.8 mS/cm [46,47].

The third analysis in the series evaluating physicochemical parameters was the assessment of honey colour, an essential attribute influencing consumer perception and indicative of botanical and geographical origin. The obtained results, expressed on the Pfund scale, revealed that the sample had a colour value of 87 mm Pfund, categorising it within the amber classification (85–114 mm Pfund) according to the United States Department of Agriculture (USDA), Agricultural Marketing Service—United States Standards for Grades of Extracted Honey (1985) [32].

This finding suggests that the analysed honey sample belongs to darker honey types, known for possessing enhanced bioactive properties. Previous studies [48] have demonstrated that darker or amber-coloured honeys typically contain higher concentrations of polyphenolic compounds compared to lighter-coloured varieties. Additionally, the antioxidant capacity of honey has been shown to significantly correlate with its phenolic content, with darker types—particularly honeydew honey—exhibiting superior antioxidant activity relative to lighter honey varieties [49,50]. These results further substantiate the hypothesis that honey colour serves as a reliable indicator of antioxidant potential, implying that darker honeys such as honeydew may confer greater health benefits owing to their higher polyphenol concentrations and improved bioactive profiles.

Free acidity, another essential parameter, directly influences the honey’s resistance to microbial spoilage. Elevated acidity acts as a natural preservative, enhancing product stability. The maximum allowable free acidity in honey is 50 meq/kg [29]. In our analysis of the LHH sample, the free acidity was found to be well within the acceptable range, measured at 35 meq/kg, which suggests that the LHH has a high-quality composition with minimal risk of spoilage. This result supports findings from previous studies that highlight free acidity as one of the most relevant indicators of honey freshness and overall quality. The inclusion of free acid in European compositional guidelines further underscores its importance in evaluating honey stability [51].

Finally, the pH of honey can influence enzymatic activity and microbial growth. Honeydew honeys typically have a higher pH than nectar honeys, with an average reported value of 4.66 [52,53]. The pH of the LHH sample was measured at 4.93, which is in line with expectations and further supports its classification as HH [52,54].

Overall, the analysed physicochemical parameters confirm that LHH meets the established quality standards for honeydew honey. Its low moisture content, high electrical conductivity, balanced acidity, and pH values are indicative of good stability, proper classification, and potential for extended shelf life.

### 3.2. Pollen Analysis

**Figure 2:**
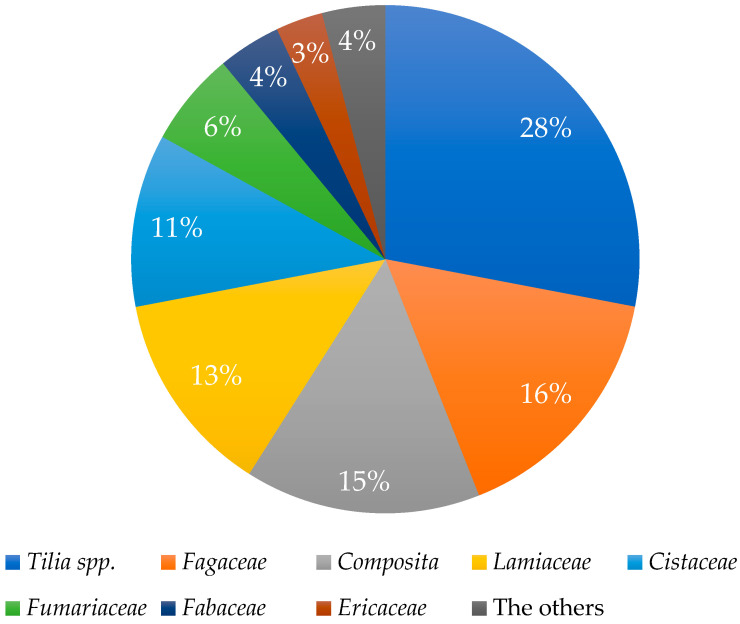
Pollen analysis characteristics of the honeydew honey sample.

Melissopalynological analysis, which involves the microscopic identification and quantification of pollen grains, serves as a valuable tool for determining the botanical origin of honey [55,56]. Although this method is somewhat less applicable to honeydew honeys—since they are derived primarily from aphid exudates rather than floral nectar—it still offers important insights into the surrounding vegetation that may indirectly influence honey composition [57,58]. In this study, the botanical origin of the LHH was confirmed through a combination of beekeeper records, geographical location data, and knowledge of dominant local flora. The melissopalynological analysis revealed a predominance of pollen from the genus *Tilia* (linden), confirming that the samples originate from areas rich in linden trees. The quantitative distribution of pollen grains within a representative sample is shown in Figure 2, further supporting the botanical authenticity of the honey under investigation.

### 3.3. Chemical Characterisation of Phenolic and Volatile Constituents

To fully uncover the complex characteristics of honey, it is essential to analyse both its phenolic and volatile components. This study aimed to perform a comprehensive chemical analysis of the LHH sample, complementing standard physicochemical and melissopalynological profiling with detailed insights into its phytochemical composition. Phenolic compounds contribute significantly to the nutritional value and antioxidant potential of honey, while volatile components play a crucial role in defining its aromatic profile and may also contribute to its antimicrobial activity. Together, these bioactive constituents serve as intrinsic markers of authenticity and origin, particularly relevant for honey derived from linden-rich environments. By identifying and quantifying the phenolic and volatile profiles of LHH, this study enhances our understanding of its overall quality, bioactivity, and botanical credibility, thereby expanding the established criteria used for its authentication and classification.

The GC-MS analysis of the LHH sample revealed a complex and diverse volatile profile, with a total of 30 identified compounds belonging to various chemical classes, including terpenes, aldehydes, alcohols, acids, and furan derivatives (Table 2). Among the detected volatiles, α-terpinolene was the most abundant compound, comprising 17.40% of the total area, suggesting that this monoterpene is a major determinant in shaping the aromatic profile of LHH. Terpinolene is commonly associated with fresh, woody, and herbal notes, and its presence is characteristic of plant species within the *Tilia* genus [59].

The analysis of volatile organic compounds (VOCs) in a unifloral LHH sample revealed a distinct chemical profile, with α-terpinolene emerging as the dominant compound (17.40%), followed by 2-furan carboxaldehyde (7.80%), and formic acid (5.24%) (Table 2). This composition underscores a significant presence of terpenes, furanone, and phenolic compounds, which collectively contribute to the unique aroma and potential bioactivity of LHH. The volatile composition of LHH reveals a distinctly different chemical fingerprint when compared to our previous analyses of chestnut and beech honeydew honeys. For example, in chestnut HH, benzaldehyde was typically the predominant compound, responsible for its characteristic sweet scent. In contrast, the aromatic profile of LHH is likely shaped by the high abundance of α-terpinolene, which is strongly associated with linden sources [17]. Other major volatiles included 2-furan carboxaldehyde (furfural) at 7.80%, a furan derivative, and formic acid (5.24%), which contributes to the acidic and sharp nuances in the honey aroma. Notably, δ-3-carene (4.01%) and hotrienol (2.99%) were also detected in relatively high amounts. These compounds are typically linked to resinous and balsamic notes, which may explain the subtle yet complex aromatic perception of LHH as observed in sensory analysis. Several low-molecular-weight compounds such as acetone (2.21%), decanal (2.09%), and 2-methylfuran (1.97%) were also identified, suggesting the presence of secondary metabolites derived from plant or microbial enzymatic processes. The identification of carvacrol (1.34%) [60,61], *p*-cymene (0.58%) [61], and thymol (1.09%) [62] indicate the presence of phenolic volatiles with known antimicrobial properties. These compounds, even in low abundance, may contribute to the bioactive potential of LHH. Overall, the volatile profile of LHH reflects a balance between terpene-dominated compounds and secondary aroma-contributing volatiles. This composition is indicative of its botanical origin from linden trees and may also contribute to its functional properties, including antioxidant and antimicrobial activity.

When compared to previously analysed chestnut and beech honeydew honeys, the volatile profile of LHH shows notable compositional differences that reflect its distinct botanical origin [17]. While benzaldehyde was found in all three honey types, it was the dominant compound in the chestnut samples and considerably more abundant in the beech honey as well, whereas in LHH it accounted for only 1.39%. Similarly, hotrienol appeared in all the samples but at markedly lower levels in LHH (2.99%) compared to chestnut honeydew honey, indicating a shared compound expressed at different intensities. Furthermore, 2-furanmethanol, which was prominent in the beech honeydew honey, was absent from the LHH sample, underscoring phytochemical divergence. On the other hand, terpenes such as α-terpinolene (17.40%) and δ-3-carene (4.01%), which were not prominent in the chestnut or beech samples, were among the most abundant volatiles in LHH, highlighting its unique, linden-specific aroma profile. Additionally, the detection of carvacrol, thymol, and *p*-cymene in LHH, compounds not reported at relevant levels in the other two honey types, further supports the differentiation of LHH as a distinct unifloral honeydew honey, both in chemical composition and potential bioactivity.

Several of the identified volatiles, particularly α-terpinolene, hotrienol, and 1,8-cineole, are commonly associated with Tilia spp. honeys and have also been reported in linden nectar honey [19,20,21]. These compounds are believed to contribute to the characteristic aroma profile of linden honey and may serve as useful markers of botanical origin. Interestingly, one of the hallmark compounds of linden nectar honey, lindenin (4-(2-hydroxypropan-2-yl)cyclohexa-1,3-diene-1-carboxylic acid), was not detected in our linden honeydew honey sample despite being consistently reported in nectar-based honeys from *Tilia* spp. [20,21]. The absence of lindenin in our honeydew sample could be explained in several ways. First, it may be that lindenin forms only during the transformation of nectar into honey inside the hive through specific enzymatic or oxidative processes that do not happen with honeydew. Second, since honeydew comes from the secretions of plant-sucking insects, its composition reflects a different biological pathway, one that likely skips the formation of this particular compound. Nevertheless, the presence of other Tilia-associated volatiles confirms the botanical link and supports the idea that both floral and honeydew honeys from linden trees may share certain aromatic features.

In addition to volatile compounds, the antioxidant and bioactive potential of honey is strongly influenced by its content of phenolic compounds and flavonoids, which are known to contribute to a variety of health-related properties, particularly antioxidant activity. HHs are generally recognised for their higher phenolic content compared to floral honeys, largely due to their darker colour, higher mineral content, and more complex chemical composition. To further evaluate the bioactive potential of LHH, spectrophotometric assays were conducted to determine total phenolic content, total flavonoid content, and antioxidant capacity using the DPPH method (Table 3). The analysis of the LHH sample determined the following values: TPC was 816.38 ± 24.84 mg GAE/kg and the total flavonoid content determined by aluminium chloride was 245.21 ± 10.57 mg QE/kg. In contrast, the total flavonoid content determined by the method with 2,4-dinitrophenylhydrazine was 18.21 ± 3.02 g NAR/kg. The antioxidant activity, measured by the DPPH method, is 1.11 ± 0.02 mmol TE/kg (Table 3). These findings suggest that LHH has significant antioxidant potential, which is consistent with previous studies showing that HH generally exhibits more potent antioxidant activity compared to nectar-based honeys, primarily due to its elevated levels of polyphenols and minerals [63]. Furthermore, the high TPC and TFC values observed in this study are consistent with other research showing that dark honeys, such as HH, tend to have higher phenolic content and, thus, higher antioxidant capacity than lighter varieties [13].

When compared to our previous results on chestnut and beech HHs [17], the antioxidant activity of LHH, as measured by the DPPH assay, was lower but still considerable. Specifically, the beech HH samples previously analysed exhibited higher DPPH values ranging from 1.48 ± 0.06 to 1.81 ± 0.18 TE/kg, while LHH showed a value of 1.11 ± 0.02 mmol TE/kg [17]. This variability highlights the influence of botanical origin on the antioxidant potential of different HHs. Such differences likely arise from the distinct phenolic profiles and associated bioactive compounds contributed by the surrounding flora.

These findings are in agreement with other studies showing that dark honeys, including buckwheat and heather, are typically rich in phenolic compounds and possess strong antioxidant properties, similar to those observed in some HH types [13]. Overall, these data support the existing literature that emphasises the value of including natural antioxidants such as those found in bee products in the diet for improved health benefits against conditions associated with oxidative stress [64,65].

However, it is important to acknowledge that this study was based on a single representative sample of LHH, which limits the generalisability of the findings. While the results provide meaningful insights and complement our previous research on chestnut and beech HHs [17], further investigations using larger and more diverse sample sets are essential to fully characterise the bioactive potential and compositional diversity among different types of honeydew honey. The observed distinctions among HH types suggest that the targeted use of specific honey varieties could be optimised based on their chemical and functional properties.

### 3.4. Biological Activity

Given its complex chemical composition and richness in phenolic compounds, honeydew honey has been increasingly recognised as a multifunctional natural product with diverse biological properties. While its antioxidant potential has been well documented, there remains a need to further explore and characterise its effects on microbial pathogens, mammalian cell viability, and tissue regeneration. The aim of this section was to evaluate the biological activity of the LHH sample through a series of in vitro assays, including antimicrobial testing, cytotoxicity screening on human keratinocytes, and a scratch assay to assess its regenerative potential.

#### 3.4.1. Antimicrobial Activity

Building on the observed antioxidant potential and rich phenolic composition of LHH, its antimicrobial activity was further evaluated to explore its potential application as a natural antibacterial agent. Honeydew honeys are particularly recognised for their ability to inhibit the growth of various pathogens, primarily due to their high content of phenolic compounds, low pH, osmotic pressure, and bioactive volatiles.

**Figure 3:**
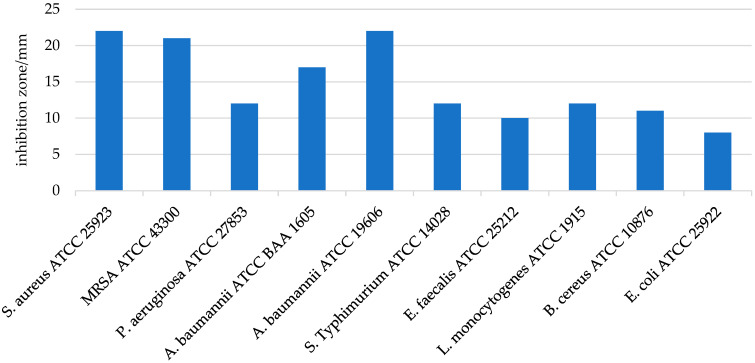
Inhibition zones (mm) of linden honeydew honey (LHH) sample against different bacteria. Data are presented as mean ± SD (*n* = 3).

The antimicrobial activity of LHH was tested in vitro against several relevant bacterial strains. The results demonstrated significant efficacy against Gram-positive bacteria, with the highest zones of inhibition observed for *S. aureus* (22.0 ± 0.0 mm) and MRSA ATCC (21.0 ± 0.5 mm) (Figure 3). These findings are in line with previous studies showing that LHH exhibits potent antimicrobial properties, especially against Gram-positive pathogens such as *S. aureus* [66,67]. In contrast, LHH showed lower activity against Gram-negative bacteria, with inhibition zones of 12.0 ± 0.0 mm for *P. aeruginosa* and 8.0 mm for *E. coli*, indicating a reduced effect on these organisms [18]. This differential sensitivity is commonly attributed to structural differences between Gram-positive and Gram-negative bacterial cell walls; Gram-negative bacteria possess an outer membrane that limits the penetration of hydrophobic antimicrobial compounds, such as flavonoids and phenolic acids, which tend to act more effectively on the more permeable peptidoglycan layer of Gram-positive bacteria [18]. To further validate these findings, the minimum inhibitory concentration (MIC) and minimum bactericidal concentration (MBC) values were determined (Table 4). The MIC values confirmed the high sensitivity of *S. aureus* and MRSA to LHH, with values of 0.025 g/mL and 0.0125 g/mL, respectively. In contrast, the MIC values for Gram-negative strains such as *P. aeruginosa* were considerably higher, exceeding 0.1 g/mL. These results corroborate earlier studies highlighting the potent antimicrobial activity of honeydew honeys, in some cases approaching that of well-known therapeutic honeys such as manuka honey [9,68,69].

A recent comparative study by Hulea et al. (2022) [70] demonstrated that linden nectar honey showed moderate antimicrobial activity, ranking lower than manuka and brassica honeys. In contrast, our LHH sample exhibited a strong inhibition of *S. aureus*, including MRSA, suggesting that honeydew-derived varieties may possess enhanced bioactivity, possibly due to higher polyphenol and mineral content.

#### 3.4.2. Assessment of Cytotoxicity (LC_50_)

Given the growing interest in the therapeutic and topical application of honey-based products, the assessment of their cytotoxic potential is essential for establishing both efficacy and safety. While honeydew honeys are known for their rich bioactive composition, including high levels of phenolics and flavonoids, these same compounds—despite their well-documented health benefits—may exert cytotoxic or pro-oxidative effects at elevated concentrations. Therefore, to evaluate the biocompatibility of LHH, its cytotoxic effects were assessed using the XTT assay on HaCaT human keratinocyte cell lines, a well-established in vitro model for testing skin-relevant safety profiles.

**Figure 4:**
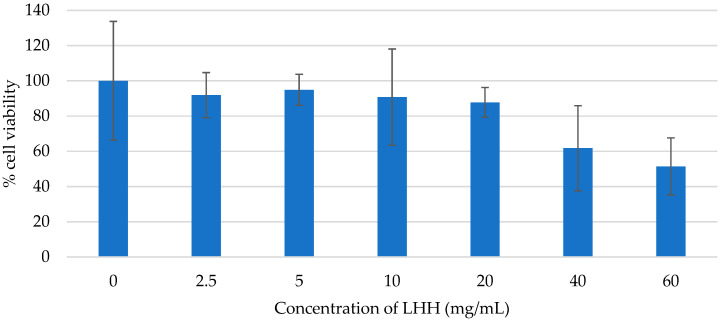
Cell viability of the HaCaT keratinocytes treated with the linden honeydew honey (LHH), assessed using the XTT assay. The percentage of cell viability was measured for different concentrations of honey (expressed in mg/mL of culture medium; range: 0–60 mg/mL). Data are presented as mean ± SD (*n* = 3).

The results revealed no significant reduction in cell viability across the tested concentration range (2.5–60 mg/mL), indicating that LHH does not exhibit acute cytotoxicity under the applied experimental conditions (Figure 4). Importantly, none of the tested concentrations reached the LC_50_ threshold, suggesting that LHH maintains a favourable cytotoxicity profile within this dose range. These findings are consistent with previously published studies, which reported high tolerance of HaCaT cells to various phenolic extracts and biocidal agents at similar or higher concentrations [71,72,73]. For instance, phenolic compounds are typically considered cytotoxic only at concentrations between 40 and 160 μg/mL, while biocides must exceed 0.02% to induce a measurable reduction in HaCaT viability.

Although the tested LHH concentrations demonstrated no detectable cytotoxicity, the absence of an LC_50_ should not be interpreted as definitive evidence of complete safety, particularly at concentrations exceeding 60 mg/mL or in cases of prolonged exposure. Further studies, including time-dependent assays and evaluations of potential sublethal effects, are warranted to refine the safety profile of LHH and determine its long-term impact on keratinocyte function and viability.

#### 3.4.3. Wound Healing Assay

Wound healing is a dynamic and multifactorial biological process involving the coordinated activity of various cell types, signalling molecules, and growth factors, ultimately leading to the restoration of skin structure and function [74]. In vitro models employing cultured fibroblasts or keratinocytes have become widely accepted for preliminary evaluation of the wound-healing potential of natural products, including plant extracts and pharmaceutical agents, particularly through their effects on cell proliferation and migration [75,76].

Honey significantly influences wound healing through various mechanisms. Studies have shown that honey accelerates wound healing [77] and promotes dermal repair, epithelialisation, angiogenesis, and immune response while reducing healing-related infections [78]. These biological activities have led to the increasing incorporation of honey into clinical wound management strategies [79]. Although honey is associated with beneficial wound-healing potential, wound healing assays are not commonly performed in studies investigating various honey characteristics.

**Figure 5:**
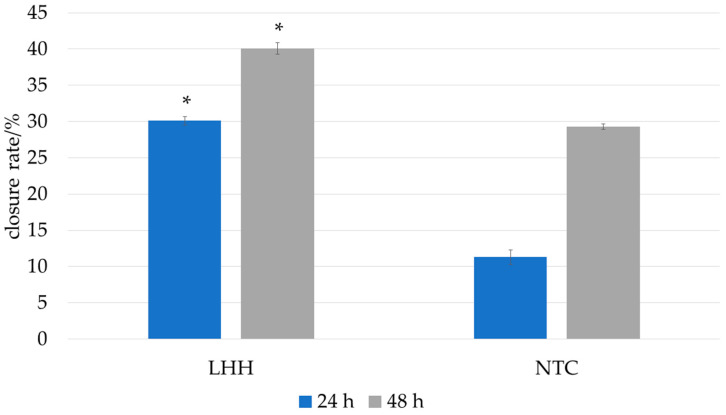
Influence of the linden honeydew honey (LHH) on the in vitro wound-healing rate 24 h and 48 h period after wounding. The wound-healing rate was expressed as the percentage of scratch closure relative to the initial scratch area. The model wounds were treated with 0.1% honey, and serum-free DMEM served as the control. Data are expressed as the mean ± SD (*n* = 4). The asterisk (*) represents statistical significance obtained with a two-tailed Mann–Whitney test between the honeydew honey sample and non-template control (NTC) at two time points and significance level *p* = 0.05.

**Figure 6:**
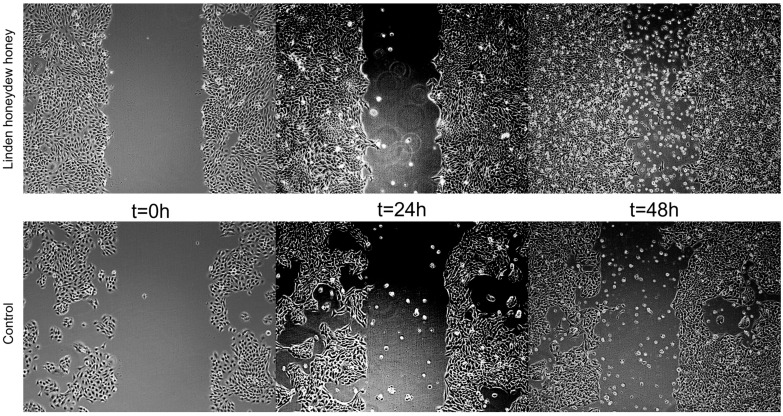
Representative micrographs of untreated HaCaT cell model wounds (control) and model wounds treated with LHH for 24 and 48 h.

In the present study, the scratch wound healing assay was performed using human HaCaT keratinocytes to assess the potential of LHH to promote epithelial regeneration. LHH treatment demonstrated a positive effect on cell migration and scratch closure, indicating its potential to support epithelial wound repair. These findings are consistent with those of Martinotti et al., who similarly observed enhanced wound-healing effects following treatment with honeydew honey in vitro [10]. The wound-healing effect of LHH is illustrated in Figure 5 and Figure 6, which show the progression of scratch closure at 24 and 48 h post-injury. The wound-healing rate was expressed as the percentage of scratch closure relative to the initial scratch area, confirming the pro-regenerative activity of LHH under the applied conditions.

The biological activity assays conducted in this study confirmed that LHH exhibits a broad spectrum of beneficial effects. The honey demonstrated strong antimicrobial activity, particularly against Gram-positive bacteria such as *Staphylococcus aureus* and MRSA, with lower efficacy against Gram-negative strains. The MIC and MBC values supported these findings, positioning LHH among honeydew honeys with relevant antibacterial potential. The cytotoxicity assessment using the XTT assay on HaCaT keratinocytes showed no significant toxicity within the tested concentration range (2.5–60 mg/mL), suggesting that LHH is biocompatible and safe for topical application under these conditions. Furthermore, the in vitro wound healing assay indicated that LHH promotes keratinocyte migration and scratch closure, highlighting its potential in epithelial regeneration and wound care. Taken together, these results point to LHH as a promising natural agent with antimicrobial, regenerative, and non-cytotoxic properties, warranting further investigation in the context of therapeutic and dermatological applications.

### 3.5. Strengths and Limitations of the Study

This study presents several key strengths: First, it employs advanced analytical techniques, including GC-MS and bioassays for antimicrobial, cell viability, and wound-healing properties, enabling a comprehensive assessment of the bioactive potential of HH. The combination of physicochemical characterisation with in vitro biological assays provides a deeper understanding of the therapeutic properties of HH beyond its basic compositional profile. Notably, the study includes unifloral LHH samples, which are particularly difficult to obtain due to the highly specific ecological conditions required for their production. Unlike nectar honeys, HHs rely on the excretions of sap-feeding insects, a process influenced by the availability of host trees, seasonal dynamics, and climatic conditions.

Furthermore, by incorporating multiple biological activity assays, including viability on keratinocytes (HaCaT) and wound-healing potential, this study expands upon previous research that has primarily focused on antioxidant and antimicrobial properties. These additional bioassays provide new insights into the therapeutic relevance of honeydew honey, supporting its potential application in dermatological and wound-care treatments.

Despite these strengths, certain limitations should be acknowledged. The sample size, while representative of unifloral LHH, remains small due to the challenges associated with obtaining pure HH from a single botanical source. Future studies should prioritise expanding the dataset by including HHs from multiple harvest seasons and regions to assess the consistency and variability of their bioactive properties.

Another limitation is the lack of in-depth analysis of environmental factors such as seasonal variation and geographic location, which may influence the phenolic and volatile profiles of HH. These factors could play a critical role in determining the honey’s chemical composition and should be considered in future research to refine HH classification. Additionally, while the study compares HH to the existing literature, a broader comparative analysis with nectar honeys from the same botanical sources would further enhance the understanding of their compositional and functional differences.

## 4. Conclusions

This study provides a comprehensive characterisation of linden honeydew honey (LHH) from Bosnia and Herzegovina, a variety that previously lacked detailed analysis. Physicochemical analyses confirmed its classification as honeydew honey, with notable characteristics including high electrical conductivity and low moisture content. The volatile compound analysis revealed α-terpinolene as the dominant compound, distinguishing LHH from other honeydew honey types. Furthermore, LHH exhibited a high total phenolic content and moderate antioxidant capacity. Antimicrobial testing demonstrated strong activity against *Staphylococcus aureus*, MRSA, and different Gram-negative bacteria, along with promising wound-healing properties observed in vitro using HaCaT keratinocytes, without any cytotoxic effects. These findings suggest that LHH possesses significant bioactive properties. While these results are promising, further research is necessary. Future studies should include a broader range of LHH samples to fully explore the variability in composition and to confirm the therapeutic relevance of LHH compared to other types of honeydew honey.

## Figures and Tables

**Table 1:** Physicochemical characteristics of linden honeydew honey (LHH) sample.

Physicochemical Parameter	LHH	Reference Values
Water (%)	15.10	≤20% [30]
Electrical conductivity (ms/cm)	1.21	≥0.8 mS/cm [30]
Free acidity (meq/kg)	35	≤50 [30]
pH	4.93	4.5–6.5 [42]

**Table 2:** Linden honeydew honey (LHH) volatile compounds analysed via GC-MS.

Compound	LHH	
	RI ^1^	Area (%)
Ethanol	<900	1.00
Aceton	<900	2.21
Dimethyl sulphide	<900	1.43
Formic acid *	<900	5.24
2-Methylfuran *	<900	1.97
Dimethyl disulfide	<900	0.67
Methylbenzene (Toluene)	<900	0.66
2-furan carboxaldehyde (furfural) *	<900	7.80
2-Furanmethanol	<900	0.59
2-acetylfuran	914	0.42
Benzaldehyde	965	1.39
Hexanoic acid	974	0.18
Octanal	1006	0.31
δ-3-carene	1016	4.01
p-cimen	1028	0.58
Benzyl alcohol	1037	0.57
2-Phenylacetaldehyde	1048	1.30
Trans-Linalool oxid	1076	0.33
α-terpinolene	1090	17.40
Linalool	1101	0.93
Hotrienol	1106	2.99
2-Phenylethanol	1116	1.61
Phenylacetonitrile	1140	0.18
2,3-Dihydro-2,5-dihydoxy-6-methyl-4-H-pyran-4-one *	1144	0.28
Methyl salicylate	1195	0.11
Decanal	1207	2.09
5-Hydroxymethylfurfural	1230	0.74
4-(1-methylethyl)-benzaldehyde	1243	0.32
Nonanoic acid	1273	0.59
Carvacrol	1300	1.34
Thymol	1303	1.09

^1^ RI—retention indices on HP-5MS column. * connection identified by condition (just by the analysis of the mass spectra).

**Table 3:** Spectrophotometric determination of total phenolic content (TPC), total flavonoid content (TFC), and antioxidant activity (DPPH) in linden honeydew honey (LHH) sample.

Analysis	LHH
TPC (mg GAE ^1^/kg)	816.38 ± 24.84
TFC-Al (mg QE ^2^/kg)	245.21 ± 10.57
TFC-DNP (g NAR ^3^/kg)	18.21 ± 3.02
DPPH (mmol TE ^4^/kg)	1.11 ± 0.02

The results are expressed as a mean ± SD (*n* = 3). ^1^ GAE—gallic acid equivalents; ^2^ QE—quercetin equivalents; ^3^ NAR—naringenin equivalents; ^4^ TE—Trolox equivalents.

**Table 4:** Minimum inhibitory concentrations (MICs ^1^) and minimum bactericidal concentrations (MBCs ^2^) of linden honeydew honey (LHH) sample and antibiotic controls (Vancomycin and Meropenem) against selected bacterial strains.

	Honey Sample		Antibiotic Control			
Bacteria	LHH		Vancomycin		Meropenem	
	MIC	MBC	MIC	MBC	MIC	MBC
*S. aureus* ATCC 25923	0.0125	0.0125	10^−6^	10^−6^	ND *	ND
MRSA ATCC 43300	0.0125	0.0125	5 × 10^−7^	10^−6^	ND	ND
*P. aeruginosa* ATCC 27853	0.1	0.1	ND	ND	6.4 × 10^−8^	6.4 × 10^−8^
*E. coli* ATCC 25922	0.05	0.1	ND	ND	6 × 10^−9^	6 × 10^−9^
*A. baumannii* 19606	0.05	0.05	ND	ND	>3.2 × 10^–5^	>3.2 × 10^–5^
*S. Typhimurium* ATCC 14028	0.1	0.1	2.0 × 10^−6^	2.0 × 10^−6^	ND	ND
*E. faecalis* ATCC 25212	0.1	0.2	1.0 × 10^−6^	1.0 × 10^−6^	ND	ND
*L. monocytogenes* ATCC 1915	0.1	0.1	1.0 × 10^−6^	1.0 × 10^−6^	ND	ND
*B. cereus* ATCC 10876	0.1	0.1	2.0 × 10^−6^	2.0 × 10^−6^	ND	ND

The results are expressed in g/mL. ^1^ MIC—concentration (g/mL) required for 99% bacteriostatic effect; ^2^ MBC—concentration (g/mL) required for 99% bacterial killing effect; * ND—Not determined.

## Data Availability

The original contributions presented in this study are included in the article. Further inquiries can be directed to the corresponding author.
